# Prenatal Identification of an EDA Variant in Dichorionic Male Twins: CfDNA Signal with Invasive Confirmation

**DOI:** 10.3390/genes16121484

**Published:** 2025-12-10

**Authors:** Simone Marcella, Roberto Sirica, Nadia Petrillo, Monica Ianniello, Alessio Mori, Rosa Castiello, Sossio Federico Capone, Eloisa Evangelista, Teresa Suero, Raffaella Ruggiero, Alfredo Columbro, Antonio Barone, Ioannis Malandrenis, Antonio Fico, Giovanni Savarese

**Affiliations:** 1 Ames Centro Polidiagnostico Strumentale S.r.l., Via Padre Carmine Fico 24, 80013 Casalnuovo di Napoli, Italy; roberto.sirica@centroames.it (R.S.); nadia.petrillo@centroames.it (N.P.); monica.ianniello@centroames.it (M.I.); alessio.mori@centroames.it (A.M.); rosa.castiello@centroames.it (R.C.); caponefederico@gmail.com (S.F.C.); eloisa.evangelista@centroames.it (E.E.); teresa.suero@centroames.it (T.S.); raffaella.ruggiero@centroames.it (R.R.); alfredo.columbro@centroames.it (A.C.); antonio.barone@centroames.it (A.B.); centroames@libero.it (A.F.); giovanni.savarese@centroames.it (G.S.); 2 Ospedale Isola Tiberina—Gemelli Isola, Via di Ponte Quattro Capi, 6, 00186 Roma, Italy; ioannismalandrenis@virgilio.it

**Keywords:** XLHED, *EDA* gene, ectodermal dysplasia, Fc-EDA, prenatal therapy, hypohidrosis, NGS

## Abstract

Background/Objectives: X-linked hypohidrotic ectodermal dysplasia (XLHED) is a rare monogenic disorder characterized by hypohidrosis, hypotrichosis, and hypodontia, caused primarily by pathogenic variants in the *EDA* gene. XLHED predominantly affects males due to its X-linked recessive inheritance, while female carriers may exhibit variable phenotypes due to random X-inactivation. Early diagnosis is critical for timely counseling and emerging therapeutic interventions. We report a rare prenatal diagnosis of XLHED in dizygotic dichorionic male twins during a dichorionic diamniotic pregnancy. At 24 weeks’ gestation, ultrasonographic anomalies—facial dysmorphisms, oligodontia, and hypoechogenic skin—raised suspicion for ectodermal dysplasia. Methods: Non-invasive prenatal test and targeted next-generation sequencing (NGS) of Cell-free DNA identified an hemizygous *EDA* deletion (c.612_629del; p.Ile205_Gly210del) with 52% variant allele frequency. Results: This in-frame deletion affects a highly conserved region in the TNF homology domain of ectodysplasin-A1, likely compromising protein function. The variant was confirmed in both fetuses via genetic analysis on amniotic fluid and in the heterozygous state in the mother, consistent with X-linked recessive inheritance. Family history revealed a maternal uncle with XLHED. Additional heterozygous variants were also identified in *CPT2*, *GBA1*, *GJB2*, and *SMN1* genes. Following comprehensive genetic counseling, the mother opted for abortion. Conclusions: This case underscores the value of applying advanced genomic technologies—cfDNA-based NGS—for prenatal diagnosis of rare genetic disorders. The identification of apathogenic *EDA* variant expands the mutational spectrum of XLHED and supports early diagnosis for informed reproductive decisions and potential access to emerging prenatal therapies. Broader application of such technologies may improve outcomes in future pregnancies at risk for monogenic disorders.

## 1. Introduction

X-linked hypohidrotic ectodermal dysplasia (XLHED) is a rare genetic disorder of ectodermal development, characterized by the triad of hypohidrosis (reduced or absent sweating), hypotrichosis (sparse hair), and hypodontia (missing or malformed teeth) [1]. The disorder predominantly affects males, given its X-linked recessive inheritance pattern, while female carriers may exhibit a variable phenotype due to the phenomenon of X-chromosome inactivation (lyonization), often leading to asymmetry or mosaic distribution of symptoms [2]. At the molecular level, XLHED is caused by pathogenic variants in the *EDA* gene, located on Xq12–q13.1 [3] encoding for ectodysplasin-A, a signaling molecule belonging to the tumor necrosis factor (TNF) superfamily [4]. Ectodysplasin-A plays a pivotal role in epithelial–mesenchymal signaling during embryogenesis, especially in the initiation and morphogenesis of ectodermal appendages such as hair follicles, sweat and sebaceous glands, and teeth [5]. The protein exists in several isoforms generated by alternative splicing, among which EDA-A1 is the most functionally relevant. EDA-A1 interacts with its receptor, EDAR, triggering a downstream cascade via EDARADD (EDAR-associated death domain) and activation of the NF-κB signaling pathway. This molecular cascade is essential for the early patterning, placode formation, and differentiation of ectodermal structures [6]. Disruption of this signaling axis—most commonly through loss-of-function mutations in *EDA* leads to failure in the development of functional sweat glands, reduced or absent dentition, and hair follicle abnormalities [7]. In the last five years, advances in high-throughput sequencing have facilitated the discovery of an expanding range of *EDA* mutations, encompassing nonsense, missense, frameshift, and splice-site variants, as well as larger-scale deletions and duplications [8,9,10,11,12]. While the correlation between genotype and phenotype remains complex, studies have shown that mutations disrupting the TNF homology domain or receptor-binding regions of *EDA* tend to result in more severe phenotypes [13]. However, inter- and intrafamilial variability suggests that modifying loci, epigenetic influences, or environmental modifiers may modulate expression [14,15,16]. Clinically, XLHED presents challenges from the neonatal period onward. Affected neonates often exhibit dry, thin skin, absent or delayed dentition, and inability to sweat, predisposing them to hyperthermia, especially in hot climates [17]. Dental anomalies are a hallmark, typically involving peg-shaped, conical, or missing teeth, which may impair feeding, speech development, and psychosocial integration [18]. Scalp and body hair are often sparse and lightly pigmented [1]. In addition to physical symptoms, psychosocial difficulties are frequently reported, including low self-esteem and social anxiety, especially during childhood and adolescence [19]. Although several studies have proposed promising and theoretically sound therapeutic strategies for XLHED, these approaches have not yet become part of standard clinical practice due to the rarity of the condition. Continued research and accumulation of clinical evidence are essential to confirm their efficacy and define effective treatment protocols.

## 2. Case Report

**Figure 1:**
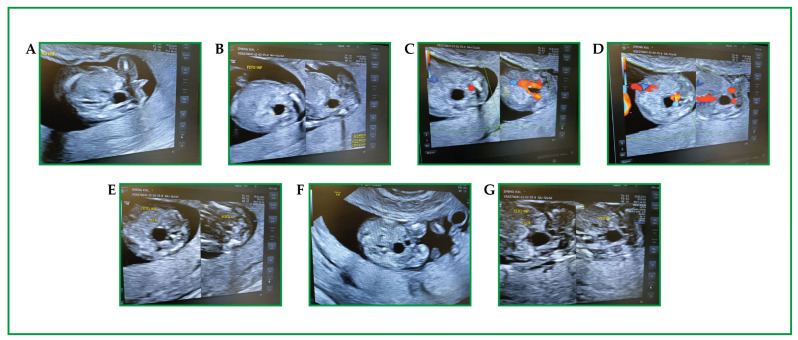
(**A**–**G**) First-trimester obstetric ultrasound in a twin pregnancy (GA 12+3): 2D and Color Doppler images focused on the fetal lower abdomen/pelvis. In both fetuses, an anechoic cystic structure is identified in the abdominopelvic region (labels “CISTI”), with smooth margins and no internal septations on the available frames. The fetal urinary bladder is visualized (labels “VESCICA”); however, based on static frames alone, the relationship between the cyst(s) and the bladder/urachus cannot be definitively established. On Color Doppler, no definite intralesional vascularity is demonstrated, while color signals are attributable to adjacent vascular structures. In one fetus, the lesion measures approximately 0.49 × 0.40 × 0.41 cm (three-axis calipers).

We report a case of a dichorionic diamniotic pregnancy. A pregnant patient of 39 years hosted two male fetuses characterized as low risk for common aneuploidies investigated via a non-invasive prenatal test (NIPT). The probands were two male fetuses referred at 24 weeks of gestation due to ultrasonographic evidence of facial dysmorphisms, oligodontia, and suspected lack of sweat glands (based on skin hypoechogenicity) (Figure 1).

**Figure 2:**
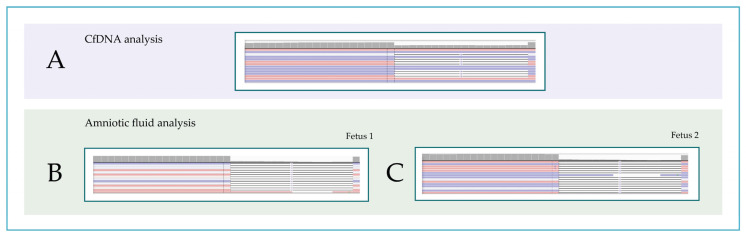
Molecular analysis of the *EDA* gene. Screenshot from Integrative Genomics View (IGV) representation of the c.612_629del p.Ile205_Gly210del mutation in the *EDA* gene identified with NGS. VAF calculated on affected cfDNA (**A**) and on amniotic fluid of fetus 1 (**B**) and fetus 2 (**C**). VAF, variant allele frequency. Red and blue lines represent the aligned reads in paired-end.

Intrauterine growth parameters were within normal range. We hypothesized that the patient was affected by a severe form of ectodermal dysplasia (ED). To confirm clinical suspicion of ED disease, we performed next-generation sequencing (NGS) on circulating maternal cfDNA. A total of 10 mL sample of peripheral blood was collected from the mother using Streck blood collection tubes (Streck tubes; Streck, Biomedical Diagnostics, Antwerp, Belgium). The samples were centrifuged at 1600× *g* for 10 min at 4 °C to separate the plasma fraction from the cellular components. Cell-free DNA (cfDNA) was isolated from 900 µL of maternal plasma using the QIAamp^®^ Circulating Nucleic Acid Kit (Qiagen, Hilden, Germany), while genomic DNA was extracted with the QIAamp^®^ DNA Blood Mini Kit, according to the manufacturer’s protocols. The obtained cfDNA was then processed for non-invasive prenatal testing (NIPT). The NIPT workflow included automated plasma processing, cfDNA purification, and library preparation using the VeriSeq NIPT Solution v2 (Microlab STAR, Illumina, San Diego, CA, USA). Sequencing was performed on an Illumina NextSeq550Dx system, following the instructions detailed in the VeriSeq NIPT Solution v2 package insert (available online at Illumina Support, https://support.illumina.com/, accessed on 1 July 2024). The concentration and quality of the extracted DNA were evaluated using spectrophotometric and fluorometric methods, providing precise quantification of cfDNA yield and purity. This analysis utilized the VERA Revolution custom gene panel, which includes 1069 genes and 100 copy number variations (CNVs) associated with monogenic disorders of significant clinical relevance and notable public health impact [20]. These genes encompass autosomal dominant, autosomal recessive, and X-linked inheritance patterns. The panel was specifically designed to improve prenatal screening, with the goal of optimizing pregnancy management and enabling early postnatal intervention. Cell-free DNA (cfDNA) was amplified and repaired using the KAPA HyperPrep Kit (Kapa Biosystems, Roche Diagnostics, Wilmington, MA, USA), following the steps of end repair, A-tailing, adapter ligation, and enrichment of DNA fragments to generate the final library for next-generation sequencing (NGS). Given the naturally fragmented nature of cfDNA, no additional fragmentation was required for the sample extracted from maternal plasma, in accordance with the KAPA HyperPrep protocol. DNA quantification was performed using a Qubit 3.0 Fluorometer with the Qubit dsDNA High Sensitivity Assay Kit (Thermo Fisher Scientific, Waltham, MA, USA), based on the fluorescent dye method. Sequencing was carried out on the NovaSeq 6000 platform (Illumina Inc., San Diego, CA, USA), achieving a mean coverage depth of at least 600×. Bioinformatic analyses were performed using in-house pipelines. In addition, genomic DNA was extracted from amniotic fluid and maternal blood for comparative analysis. NGS identified an hemizygous variant in the *EDA* NM_001399.5 c.612_629del p.Ile205_Gly210del with a VAF of 52% in cfDNA (Figure 1A). The variant introduces an in-frame deletion known for causing impaired activity, consistent with a loss-of-function mechanism. Parental testing confirmed maternal heterozygosity and paternal wild-type status, in accordance with an X-linked recessive mode of inheritance. The variant was confirmed by Sanger sequencing. Family history revealed a maternal uncle affected by XLHED. Genetic counseling prompted molecular analysis. Sweat test confirmed anhidrosis on the pregnant patient’s uncle. Then we confirmed the same mutation on amniotic fluid and we performed analysis on genomic DNA (gDNA) and we performed a test using KAPA HyperPlus kit that, differently from the KAPA HyperPrep kit used for cfDNA, included gDNA fragmentation to achieve the size required by the applied Illumina short-reads sequencing technology. Interestingly fetus one is carrier of mutations in following genes: *EDA*, *CPT2*, *GBA1*, and *GJB2* genes. While, fetus number 2 is carrier of mutations in the following genes: *EDA*, *CPT2*, and *SMA*. Finally, the *EDA* variant was confirmed on amniotic fluid in both twins (Figure 2B,C). The patient chose to have an interruption.

## 3. Discussion

XLHED represents the most severe subtype of hypohidrotic ectodermal dysplasia (ED) [17]. Molecular investigations have identified 64 genes and three chromosomal regions implicated in ED pathogenesis, including *EDA*, *EDAR*, *EDARADD*, *TRAF6*, *WNT10A*, and *NEMO* [21]. Variants in the *EDA* gene are detected in more than half of all HED cases. The *EDA* gene is also referred to as ED1, HED, EDA1, HED1, ODT1, XHED, ECTD1, XLHED, and STHAGX1 [22]. To date, over 100 distinct *EDA* gene variants have been reported in individuals with or without ED. Although many *EDA* mutations may represent null variants without a clear genotype–phenotype correlation, at least 82 have been classified as pathogenic mutations associated with HED, as reported in the NCBI ClinVar database (http://www.ncbi.nlm.nih.gov) and in published studies [23,24]. Among pathogenic *EDA* mutations, missense variants are the most commonly observed, followed by deletions and nonsense mutations that lead to premature truncation of the EDA protein. Additional variants include those affecting splice sites or resulting in in-frame deletions [9]. Based on structural analyses of the EDA protein and previously reported mutations, the majority of residues altered by missense variants are located within the TNF homology domain, the collagen domain, and the furin recognition sequence [25]. Therefore, we explored a case of a pregnant woman with a dichorionic diamniotic pregnancy. This case report describes a rare prenatal diagnosis of XLHED in dizygotic male twins, made possible through a combined approach involving ultrasonographic evaluation, maternal cfDNA sequencing, and molecular analysis on amniotic fluid. Firstly, by ultrasonographic evaluation we evaluated the presence of a retrovesical cyst in one of the twins. These data started all the subsequent investigations. Then, the identification of an hemizygous deletion in the *EDA* gene c.612_629del (p.Ile205_Gly210del) in both fetuses, along with maternal heterozygosity and a suggestive family history, strongly supports the diagnosis of XLHED with an X-linked recessive inheritance pattern. This finding highlights the utility of integrating advanced genomic technologies in the prenatal setting for early detection of monogenic disorders. For this purpose, we also report incidental pathogenic variants found through analysis of cfDNA. Prenatal suspicion was initially raised by ultrasonographic findings, including facial dysmorphisms, which are among the earliest detectable features of XLHED. Although anhidrosis cannot be directly assessed in utero, the observed skin hypoechogenicity raised further suspicion of ectodermal gland abnormalities. These phenotypic clues underscore the importance of targeted ultrasonographic assessment of dysmorphism in pregnancy. The application of cfDNA-based testing in this context is particularly noteworthy. While NIPT is traditionally employed for aneuploidy screening, the use of extended gene panels, such as the VERA Revolution platform, demonstrates how cfDNA can be leveraged to screen for pathogenic variants associated with monogenic conditions [20,26]. In this case, cfDNA analysis not only ruled out common chromosomal abnormalities but also guided subsequent targeted testing, leading to a definitive molecular diagnosis. The identified *EDA* variant has not been previously reported in major population; it is extremely low or absent in population, indicating that this is an ultra-rare or private variant, supporting its novelty [27]. The deletion, affecting amino acids 205 to 210 within a highly conserved region of the ectodysplasin-A1 protein, is predicted to result in loss of function through disruption of the receptor-binding domain [4]. This aligns with known genotype–phenotype correlations in XLHED, where mutations affecting the TNF homology domain are typically associated with a more severe phenotype, including complete anhidrosis and marked dental anomalies. The variant was confirmed in both fetuses and shown to be maternally inherited, to our knowledge for the first time within non-invasive prenatal analysis, consistent with the classical X-linked recessive transmission observed in XLHED families. Interestingly, additional heterozygous variants were identified in other clinically relevant genes (*CPT2*, *GBA1*, *GJB2*, and *SMA*). The identified variants, classified as pathogenic in the ClinVar database, were not further investigated, as they are located in genes associated with autosomal recessive conditions and were detected in the fetuses in a heterozygous state, thus not expected to confer a clinical risk. These findings highlight the complexity of interpreting incidental or secondary findings in prenatal genomic testing and underscore the need for cautious genetic counseling, particularly when discussing uncertain or low-penetrance variants with prospective parents. This case further emphasizes the psychosocial and clinical value of early screening. Timely identification of XLHED allows for anticipatory guidance, including counseling on thermoregulation strategies, early dental planning, and the potential eligibility for emerging therapies such as prenatal protein replacement with recombinant ectodysplasin-A1 (Fc-EDA) [28]. Notably, recent clinical trials have demonstrated that intra-amniotic administration of Fc-EDA can restore sweat gland function and prevent life-threatening hyperthermia in affected newborns [29]. Although this therapy was not administered in our case, early molecular screening opens the possibility for future in utero interventions, especially if diagnosis could be made in earlier gestational windows. Finally, we performed molecular analysis on amniotic fluid, combined with advanced bioinformatics algorithms and Sanger validation, showing the expanding role of comprehensive genomic analysis in perinatal medicine. As sequencing costs continue to decrease and analytical pipelines improve in sensitivity and specificity, such approaches are likely to become increasingly integrated into routine prenatal diagnostics, particularly for syndromes with heterogeneous or subtle prenatal phenotypes [30]. The identification of an *EDA* variant not only expands the mutational spectrum of the disease, but also underscores the value of early diagnosis for informed clinical decision-making, personalized counseling, and potential access to emerging prenatal therapies that may significantly improve postnatal outcomes. However, this case not only underscores the diagnostic potential of integrated prenatal genomics but also highlights the complex and deeply personal decisions that may follow such diagnoses. In this instance, following comprehensive genetic counseling and consideration of the potential severity of XLHED and the presence of additional variants of uncertain significance, the patient opted for termination of pregnancy. This decision reflects the importance of timely, accurate diagnosis and counseling to support informed reproductive choices, especially in the context of potentially severe or syndromic genetic conditions [31]. Advances in NGS have facilitated early diagnosis, even in utero, and carrier detection in at-risk families [32,33]. This has enhanced genetic counseling, allowing for informed reproductive decisions, and has supported the identification of female carriers who may benefit from dermatologic or dental monitoring [34]. Importantly, these technologies have also uncovered novel or de novo variants, expanding the mutational spectrum and providing critical insights into genotype–phenotype relationships [35]. The management of XLHED remains multidisciplinary, involving dermatologists, dentists, geneticists, and psychologists [36]. Supportive measures include custom dental prosthetics, hydration and temperature control, emollients for dry skin, and psychosocial support [37,38]. Looking ahead, emerging therapies such as gene editing (e.g., CRISPR-Cas9) and mRNA-based delivery systems are under preclinical evaluation [39]. These approaches aim to provide durable or permanent correction of the molecular defect, ideally via uterus or early postnatal intervention. Moreover, broader understanding of *EDA* pathway biology may offer therapeutic insights for related conditions, including certain forms of alopecia and dental dysplasia. In summary, XLHED represents a unique model for developmental biology, precision medicine, and prenatal intervention. The *EDA* gene remains central to understanding the intricate signaling networks that guide ectodermal patterning, and advances in molecular diagnostics and therapy continue to transform the outlook for affected individuals.

## 4. Conclusions

This case highlights the value of integrating advanced genomic technologies with targeted prenatal imaging for the early detection of rare monogenic disorders such as XLHED. The identification of an hemizygous *EDA* variant enabled a timely and accurate diagnosis, guiding informed reproductive decision-making. The use of cfDNA analysis and amniotic fluid testing demonstrates the growing role of NGS in prenatal care. Ultimately, this case emphasizes the importance of early genetic prenatal diagnosis and a multidisciplinary approach to support both clinical management and the psychosocial needs of affected families.

## Data Availability

The original contributions presented in the study are included in the article, further inquiries can be directed to the corresponding author.
